# Functional inactivation of MDR3 caused by a homozygous *ABCB4* missense variant leading to liver failure

**DOI:** 10.3389/fgene.2026.1802238

**Published:** 2026-04-02

**Authors:** Sophia Heinrich, Annika Behrendt, Malte Sgodda, Holger Gohlke, Bernd Auber, Amelie Stalke, Björn Hartleben, Heiner Wedemeyer, Tobias Cantz, Richard Taubert

**Affiliations:** 1 Department of Gastroenterology, Hepatology, Infectious Diseases and Endocrinology, Hannover Medical School, Hannover, Germany; 2 European Reference Network for Hepatological Diseases (ERN RARE-LIVER), Hamburg, Germany; 3 Institute for Pharmaceutical and Medicinal Chemistry, Heinrich Heine University Düsseldorf, Düsseldorf, Germany; 4 REBIRTH-AG Translational Hepatology and Stem Cell Biology, Hannover Medical School, Hannover, Germany; 5 Institute of Bio- and Geosciences (IBG-4: Bioinformatics), Forschungszentrum Jülich, Jülich, Germany; 6 Department of Human Genetics, Hannover Medical School, Hannover, Germany; 7 Institute for Pathology, Hannover Medical School, Hannover, Germany

**Keywords:** ABCB4, functional variant reclassification, MDR3 deficiency, progressive familial intrahepatic cholestasis, variant of uncertain significance

## Abstract

Progressive familial intrahepatic cholestasis (PFIC) is a rare hereditary liver disorder that is caused by defective hepatobiliary transport. Variants in ATP binding cassette 4 ( ), encoding phosphatidylcholine floppase MDR3, are a frequent cause; however, many remain classified as variants of uncertain significance (VUS), limiting molecular diagnosis. Here, we functionally characterized a previously reported homozygous *ABCB4* missense variant (c.431G>A, p.(Arg144Gln)) without experimental evidence of pathogenicity. An *in silico* analysis using the ABCB4-specific prediction tool Vasor indicated a high probability of pathogenicity (0.88). Structural modeling suggested that Arg144Gln disrupted key electrostatic interactions essential for MDR3 membrane anchoring. Immunofluorescence analyses demonstrated markedly reduced membrane localization with residual cytoplasmic retention, consistent with complete loss of protein function. In conclusion, the *ABCB4* p.(Arg144Gln) variant causes functional inactivation of MDR3 and represents a novel pathogenic mutation. Combined genetic, structural, and functional analyses are valuable tools for characterizing variants of uncertain significance in *ABCB4*-associated cholestatic liver disease.

## Introduction

PFIC refers to a group of rare, autosomal recessive liver disorders characterized by defective bile formation, leading to progressive cholestasis and, ultimately, liver cirrhosis (Pfister et al., 2022; Alam and Lal, 2022). PFIC is caused by pathogenic variants in genes encoding key hepatobiliary transport proteins, including *ATP8B1*, *ABCB11*, *ABCB4*, *NR1H4*, *MYO5B and TJP2* (Pfister et al., 2022; Degiorgio et al., 2007). These variants lead to a reduction in the number or function of canalicular transport proteins in hepatocytes, resulting in impaired bile secretion, progressive intrahepatic cholestasis, and ultimately, cirrhosis (Pfister et al., 2022).

Among PFIC subtypes, *ABCB4*-associated PFIC (type 3) is characterized by defects in MDR3. The clinical presentation is variable, complicating diagnosis and management (Degiorgio et al., 2007). Merging evidence further indicates that the genetic configuration influences disease onset, with homozygous variants being associated with a markedly earlier and more severe clinical phenotype compared with heterozygous or compound heterozygous variants (Wang et al., 2024). Emerging therapeutic strategies, such as inhibitors of the ileal bile acid transporter (IBAT), are under investigation and may offer future alternatives (Di Giorgio et al., 2025). While animal models have shown promising responses to gene therapy and synthetic *ABCB4* mRNA application (Wei et al., 2021), and orthotopic liver transplantation (OLT) remains the only curative treatment currently available.

The *ABCB4* gene encodes MDR3, a member of the ATP-binding cassette (ABC) transporter family, which plays a crucial role in phosphatidylcholine translocation across the canalicular membrane of hepatocytes (Nosol et al., 2021; Ol et al., 2020). Structurally, MDR3 consists of two transmembrane domains (TMDs) and two nucleotide-binding domains (NBDs). High-resolution structural studies have revealed that MDR3 adopts an outward-facing conformation during lipid transport, likely utilizing a “credit-card swipe” mechanism to insert phospholipids into the bile (Prescher et al., 2021). The tilt and positioning of transmembrane helices are influenced by specific anchor residues, including aromatic and charged side chains, which contribute to membrane integration and functional dynamics (Vostrikov et al., 2010). While the spectrum of *ABCB4* variants continues to expand, many variants remain unclassified and the mechanism of pathogenicity is unclear, posing challenges in diagnosis and management. Functional assays and *in silico* modeling including iPSC-based hepatic organoids have become essential tools for assessing the potential pathogenicity of these variants (Behrendt et al., 2022; Sgodda et al., 2025; Behrendt et al., 2025).

Here, we report a homozygous *ABCB4* missense variant (c.431G>A p.(Arg144Gln)) with a first association with a clinical phenotype and provide evidence for the loss of MDR3 expression and membrane localization by immunofluorescence staining.

## Methods

### Clinical data assessment

Post-transplant histology, genetic testing, and family history assessments of the index patient were conducted. Clinical examinations based on serum parameters, liver sonography, liver stiffness measurements, and human genetic analyses of all available family members (two brothers) were performed.

### Genetic testing

DNA was extracted from whole blood samples and whole-genome sequencing was performed. DNA enrichment and library preparation were performed using xGen DNA Lib Prep EZ UNI (Integrated DNA Technologies, Inc., Coralville). Sequencing was performed using an Illumina NovaSeq 6000 sequencer (Illumina, San Diego, CA, USA). Alignment was performed with the Genome Reference Consortium Human Build 37 (GRCh37) using megSAP version megSAP_ 0.2-704-g87fa023d (https://github.com/imgag/megSAP).

Variant prioritization and visualization were performed with GSvar, version ngs-bits-2024_08-35-g5b45dc95 (https://github.com/imgag/ngs-bits), Integrative Genomics Viewer (IGV), version 2.16.0, and Alamut® visual Plus, version 1.13 (Interactive Biosoftware, Rouen, France) (Robinson et al., 2011). The variant was classified according to the American College of Medical Genetics and Genomics (ACMG) Standards and Guidelines using the point system and the ClinGen Variant Classification Guidance (Tavtigian et al., 2020; Richards et al., 2015).

### Vasor

The functional impact of the p.(Arg144Gln) MDR3 variant was predicted using the VASOR (Variant Assessor of MDR3) tool, available at https://cpclab.uni-duesseldorf.de/mdr3_predictor/ (Behrendt et al., 2022). The variant was submitted to the online platform by providing the respective amino acid substitution. VASOR is a machine-learning-based tool that integrates sequence- and structure-based information specific to the MDR3 (ABCB4) protein to predict the pathogenic potential of missense variants. The prediction result categorizes each variant as either *benign* or *pathogenic* and is accompanied by a probability score.

### Immunofluorescence stainings

Immunofluorescence staining of MDR3 was used to validate and analyze the potential functionality of the gene variants. Formalin-fixed liver tissue was embedded in paraffin before 5 µm sections were prepared. The sections were deparaffinized and demasked (10 mM sodium citrate, 0.05% Tween-20, pH6.0) at 96 °C for 45 min. After slow cooling, the slides were blocked with 1% BSA in TBS for 1 h at RT. Primary antibody was added (anti-MDR3: ENZO #ALX-801-028-C125; 1:25 diluted in TBS) with 1% FBS for 2 h at RT. Slides were washed and the secondary antibody was added (Invitrogen #A21206; 1:400 diluted in TBS) for 1 h at RT. After washing, cells were counterstained with DAPI (5 ng/mL, Sigma), embedded in DAKO aqueous mounting media, and analyzed using an inverted fluorescence microscope (Olympus IX70) and CellSens software. Specimens from healthy liver tissue obtained during oncologic liver surgery served as positive controls.

## Results

### Identification of a homozygous *ABCB4* variant

A 28-year-old patient presented with acute-on-chronic liver failure and a history of cirrhosis previously suspected to be Wilson’s disease in a foreign center. The patient had been treated with the copper chelator D-penicillamine for several years; however, no biochemical, histological, or genetic evidence supported this diagnosis. Laboratory testing after admission to our center revealed markedly elevated transaminase levels (>3× ULN), hyperbilirubinemia (>31 × ULN), and strongly impaired hepatic synthesis, consistent with end-stage liver disease. Following liver transplantation (MELD 38), histological analysis of the explanted liver showed pronounced ductular and intrahepatic cholestasis without copper deposits or other features typical of Wilson’s disease (Supplementary Figure S1). These findings prompted post-transplant genetic testing, which identified a homozygous missense VUS in *ABCB4* (ENST00000265723.4: c.431G>A, p.(Arg144Gln)), initially classified as aVUS. A family evaluation revealed consanguineous parents and two clinically healthy brothers. Both brothers, aged 34 and 37 years, underwent clinical evaluation, including liver ultrasonography and liver stiffness measurements, which showed no abnormalities. Serological testing yielded normal results. However, genetic analysis confirmed that both the brothers were heterozygous carriers of the same *ABCB4* variant, supporting a recessive mode of inheritance.

### Structural analysis of the MDR3 p.(Arg144Gln) variant suggests disturbance of local interaction network

**FIGURE 1:**
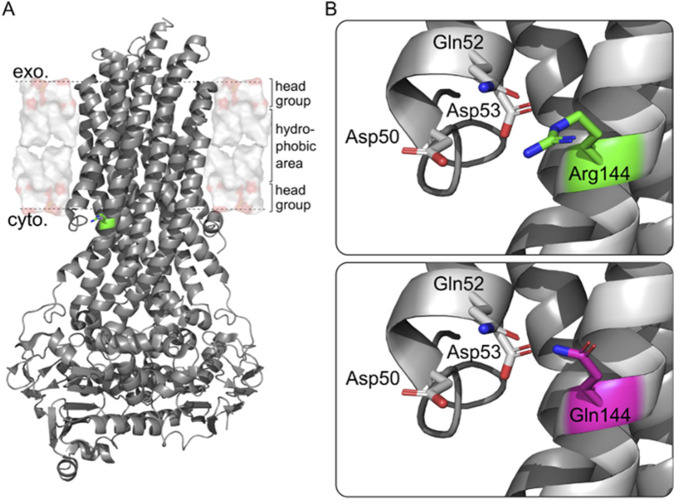
Structural analysis of the MDR3 variant Arg144Gln within the protein. **(A)** Overall MDR3 protein structure in the outward-facing conformation taken from (Prescher et al., 2021). (8) The variant site (green) is close to the membrane interface. **(B)** The side chain (shown as licorice) of the WT protein (upper panel) can form interactions with residues on the TMH1, while the shortening of the side chain of the variant protein and its neutral character (lower panel) likely decreases the interaction frequency and lowers the capability for membrane anchoring. Exo (extracellular); cyto (cytosolic).

The identified missense variant in the phosphatidylcholine floppase MDR3, p.(Arg144Gln), is located on transmembrane helix (TMH) 2, close to the membrane surface on the intracellular side (Figure 1A). The MDR3-specific prediction tool Vasor categorizes this variant as pathogenic with a probability of pathogenicity of 0.88 (range from 0, highly likely benign, to 1, highly likely pathogenic) (Behrendt et al., 2022). The positioning of the positively charged arginine residue within the protein structure suggests a membrane anchoring function, which might be impeded in the variant protein (Vostrikov et al., 2010; Sustich et al., 2020). Structural analysis of the outward-facing conformation of MDR3 further indicates an interaction network of Arg144 with residues on TMH1, namely, Asp50, Gln52, and Asp53 (Figure 1B) (Prescher et al., 2021). Of note, Gln52 has been identified as an important site for lipid transport, although there is contradicting evidence on the transportation pathway (Nosol et al., 2021; Ol et al., 2020; Prescher et al., 2021). The variant Gln144 can likely form interactions with Gln52, although the increased distance (Arg144 Cε atom–Gln52 Cδ atom: 4.4 Å; Gln144 Cδ atom–Gln52 Cδ atom: 5.7 Å, as measured in the static model with PyMol) suggests that they are weaker than in the wildtype. Thus, we hypothesized that the shortened and neutral side chain of the variant Gln144 negatively affects protein function by disturbing critical interactions with neighboring residues, in combination with a decreased capability to act as a membrane anchor.

### Functional analysis of MDR3 dysfunction

**FIGURE 2:**
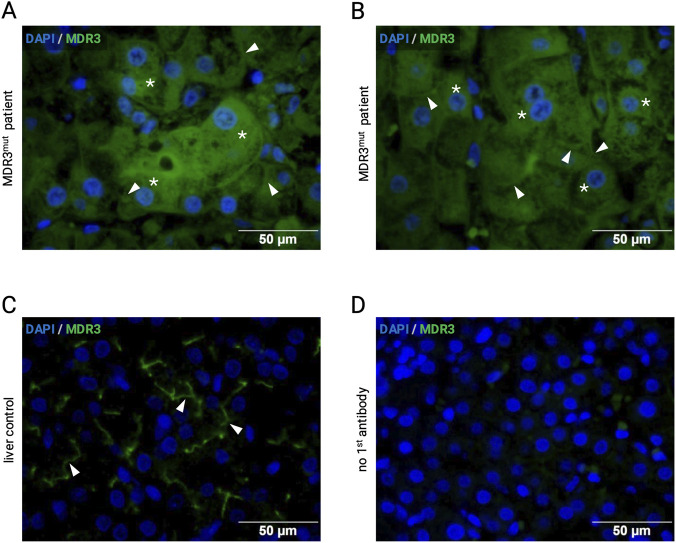
Immunofluorescence analysis of MDR3 in liver tissue sections. **(A,B)** sections from the patient’s explant liver shows predominantly cytosolic and endoplasmatic reticulum-associated MDR3 fluorescence (asterisks) with markedly reduced canalicular membrane localization (arrow heads). **(C)** control liver tissue. A liver section from a patient with metabolic liver disorders unrelated to MDR3, indicating preserved canalicular protein expression and localization. Surrounding tissue after liver tumor resection demonstrates a clear apical (canalicular) MDR3-related fluorescent signal (arrow heads). **(D)** a liver section stained only with the secondary antibody, but not with the primary antibody, served as control for the absence of relevant unspecific fluorescent signals.

To validate the prediction generated by the Vasor tool, we performed immunofluorescence staining to assess the localization and expression of the MDR3 protein (Figure 2). The variant identified (p.(Arg144Gln)) was predicted to disrupt MDR3’s membrane-anchoring and phospholipid transport functions. Consistent with this prediction, immunofluorescence staining revealed weak cytosolic and endoplasmic reticulum-associated staining of MDR3 with markedly reduced membrane localization in the patient’s native liver tissue. This aberrant pattern suggests impaired trafficking or stability of the protein in the canalicular membrane. Importantly, no staining was observed when the primary antibody was omitted, which confirmed the specificity of the signal.

As a negative control, liver tissue from two patients with metabolic liver disorders unrelated to MDR3 deficiency was analyzed. In this case, MDR3 showed strong and well-defined membrane-associated expression, indicating preserved protein localization and supporting the specificity of abnormal findings in the index case.

These results support the hypothesis that the p.(Arg144Gln) variant impairs MDR3 localization to the membrane, which is consistent with functional loss of the phospholipid transporter. By describing our homozygous patient together with the altered expression pattern in liver tissue, we were able to reclassify the variant of uncertain significance as likely pathogenic (Tavtigian et al., 2020; Richards et al., 2015) (https://clinicalgenome.org/tools/clingen-variant-classification-guidance/), which is supported by Vasor prediction and structural analysis (for variant classification before and after our study see Supplementary Table S1).

## Discussion

In this report, we describe a homozygous missense variant in the *ABCB4* gene (c.431G>A p.(Arg144Gln)), initially classified as a variant of uncertain significance, associated with the development of liver cirrhosis. Functional investigations, including *in silico* modeling and immunofluorescence studies, indicated impaired MDR3 localization and membrane integration, supporting the pathogenicity of the variant. This case highlights the importance of integrating genetic testing, protein localization studies, and structural modeling to better understand the pathomechanism and define the clinical relevance of *ABCB4* variants. Unlike PFIC types 1 and 2, which are typically present in infancy, PFIC3, due to biallelic *ABCB4* variants, can manifest from childhood to adulthood, complicating proper diagnosis (Jacquemin et al., 2001; Davit-Spraul et al., 2009). MDR3 deficiency is known for its wide clinical and histopathological spectrum (Pfister et al., 2022). The cholangiopathic injury pattern observed here, characterized by portal fibrosis, ductular proliferation, and mixed inflammatory infiltrates, corresponds to established descriptions of *ABCB4*-associated disease (Jacquemin et al., 2001). Genetic analysis suggested the presence of an underlying MDR3-related defect. Numerous *ABCB4* variants have been reported (Dröge et al., 2017), and this specific variant has been previously described only twice (Supplementary Table S1), with one report describing a patient with heterozygous p.(Arg144Gln) together with a heterozygous VUS p.(Val1078Glu), where residual MDR3 staining suggested partial protein function (Gonzales et al., 2023). Our findings now show for the first time a markedly reduced MDR3 expression and membrane localization in a patient that is homozygous for ABCB4 p.(Arg144Gln).

Arg144 resides within TMH 2 near the cytoplasmic interface (Vostrikov et al., 2010; Sustich et al., 2020). Structural modeling indicates that substitution by glutamine alters the electrostatic interactions essential for protein stability within the membrane. VASOR, an *ABCB4*-specific prediction tool, classified this variant as pathogenic (probability = 0.88), and immunofluorescence staining confirmed reduced canalicular MDR3 localization in the patient’s liver (Behrendt et al., 2022). Together, these findings strongly suggest a functionally deleterious effect leading to impaired phosphatidylcholine transport, a hallmark of PFIC3 (Davit-Spraul et al., 2010; Ruetz and Gros, 1994). This study provides further insight into the potential pathomechanism of the variant and exemplifies how combined computational and experimental approaches can reclassify variants of uncertain significance.

Segregation analysis revealed two heterozygous asymptomatic carriers, consistent with an autosomal recessive inheritance. One sibling with an unknown ABCB4 genotype died of liver disease. Previous misclassification of Wilson’s disease highlights the diagnostic challenges of rare liver disorders, as copper accumulation may also occur in cholestatic conditions. Genetic testing remains a definitive diagnostic tool that expands our understanding of pathogenic variants with clinical relevance.

These findings underline the phenotypic variability and clinical complexity of MDR3 deficiency and support the value of genetic counseling and family screening.

These findings have therapeutic implications. Standard management of advanced MDR3 deficiency remains OLT; however, emerging approaches such as IBAT inhibitors, synthetic bile acid derivatives, compounds targeting bile acid synthesis via the FXR/FGF axis, or chaperones that enhance residual activity of MDR3 show promising data in preclinical or early clinical studies. For IBAT inhibitors, even in real-life cohorts (Di Giorgio et al., 2025). Thus, early structural and functional variant characterization will be essential for implementing precision medicine strategies to identify patients who may benefit from targeted interventions before disease progression in order to delay or circumvent OLT as a final option in advanced stages of the disease.

Our study has several limitations. Functional characterization was limited to immunofluorescence of patient tissue, which does not directly quantify phosphatidylcholine transport activity. Furthermore, it does not allow us to establish a direct causal relationship between the detected variants and alterations observed in the liver tissue. However, despite the absence of functional transport assays, the complete loss of membrane localization shown by immunofluorescence analysis is highly correlated with total loss of function as described before (Delaunay et al., 2009; Park et al., 2016). An additional limitation lies in the structural modeling being based on static conformations, whichmay not fully capture the dynamic effects of the p.(Arg144Gln) substitution. Nevertheless, the convergence of genetic, structural, and experimental findings strongly supports the pathogenic nature of the variant and justifies the reclassification from VUS to likely pathogenic.

In conclusion, *ABCB4* c.431G>A p.(Arg144Gln) represents a newly validated likely pathogenic variant that is highly suspicious for causing loss of MDR3 function. This study underscores the utility of combining genetic, structural, and immunofluorescence analyses to establish the pathogenicity of *ABCB4* variants with uncertain significance. Expanding the functional annotation of *ABCB4* variants is critical for improving the diagnostic accuracy, guiding individualized management, and informing future therapeutic strategies for PFIC3 and related cholestatic diseases.

## Data Availability

The raw data supporting the conclusions of this article will be made available by the authors upon reasonable request.
