# Supplementary figures and images for: Functional inactivation of MDR3 caused by a homozygous ABCB4 missense variant leading to liver failure
Source: Front Genet. 2026 Apr 2;17:1802238. doi: 10.3389/fgene.2026.1802238 (PMC13082754; doi:10.3389/fgene.2026.1802238)

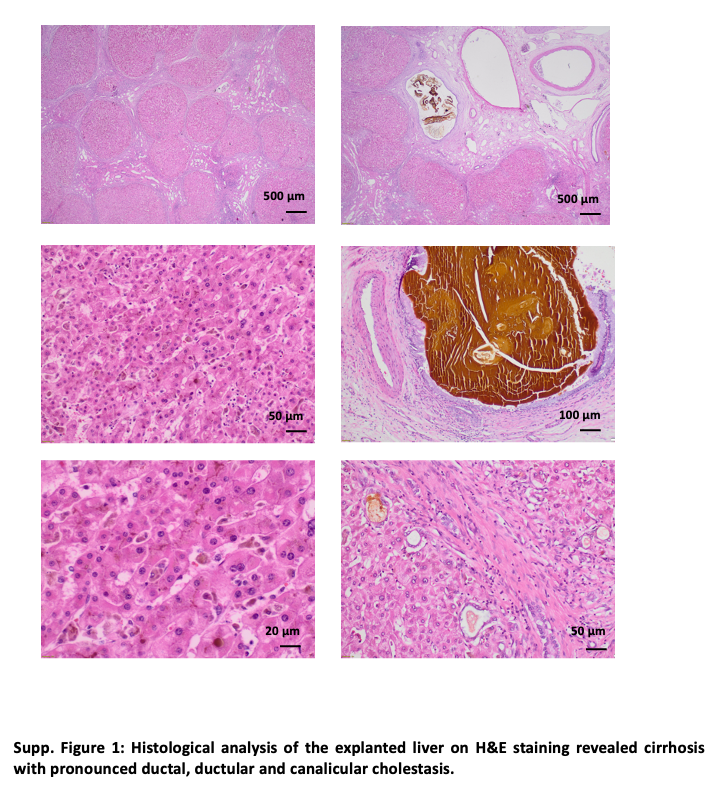

Supplement: Supplementary file 1 [file Image1.tiff]
