# Supplementary material for: Functional inactivation of MDR3 caused by a homozygous ABCB4 missense variant leading to liver failure
Source: Front Genet. 2026 Apr 2;17:1802238. doi: 10.3389/fgene.2026.1802238 (PMC13082754; doi:10.3389/fgene.2026.1802238)
Supplement: Supplementary file 2 [file Table1.docx]

**Table S1: variant classification before and after our study**

| **Gene** | ***ABCB4* (**[ENST00000265723.4](https://grch37.ensembl.org/Homo_sapiens/Transcript/Summary?db=core;g=ENSG00000005471;r=7:87031013-87109751;t=ENST00000265723)**)** |
| --- | --- |
| **Variant** | c.431G>A p.(Arg144Gln) |
| **gnomAD v4.1 (1) Filtering Allele frequency (FAF)** | 0.0007074% (PM2_supp) |
| **ClinVar (2)** | VUS (SCV001137411.1 and SCV000862499.2) |
| **REVEL-Score (3)** | 0,776 (PP3_mod) |
| **Vasor-Score (4)** | 0,88 |
| **Splicing prediction*** | unremarkable |
| **Missense gnomAD constraint Z-Score (PP2 if >3,09)** | 3,56 |
| **Literature** | 1. description of one PFIC patient heterozygous for c.431G>A and heterozygous for c.3233T>A p.(Val1078Glu)(VUS), decreased MDR3 canalicular staining (5)  2. description of one PFIC patient homozygous for c.431G>A (6)(PM3_supp) |
| **ACMG criteria**^#^ | PM2_supp, PP3_mod, PM3_supp, PP2 (5 points) |
| **Classification before our report^#^** | Variant of uncertain significance (VUS) |
| **Our study and patient description** | |
| **Zygosity of our patient** | Homozygous (together with case from (6): PM3_mod) |
| **Segregation analysis** | 2 asymptomatic heterozygous siblings |
| **MDR3 Immunofluorescence staining** | markedly reduced MDR3 membrane localization with residual cytoplasmic retention (PP4_supp) |
| **Structural *in silico* analysis** | shortened and neutral side chain of the variant Gln144 may negatively impact protein function via disturbance of critical interactions to neighboring residues, in combination with a decreased capability to act as a membrane anchor |
| **ACMG criteria^#^** | PM2_supp, PP3_mod, PM3_mod, PP2, PP4_supp (7 points) |
| **(Re-) Classification**^#^ | Likely pathogenic |

*according to Splice AI(7) and MaxEntScan(8); ^#^Variant classification according to the current ACMG Standards and Guidelines using the point system and the ClinGen Variant Classification Guidance (9-11). ACMG: American College of Medical Genetics and Genomics, VUS: variant of uncertain significance

REFERENCES

1. gnomAD - Genome Aggregation Database [Available from: <https://gnomad.broadinstitute.org/>.

2. ClinVar. ClinVar [Available from: <https://www.ncbi.nlm.nih.gov/clinvar/>.

3. Ioannidis NM, Rothstein JH, Pejaver V, Middha S, McDonnell SK, Baheti S, et al. REVEL: An Ensemble Method for Predicting the Pathogenicity of Rare Missense Variants. Am J Hum Genet. 2016;99(4):877-85.

4. Behrendt A, Golchin P, König F, Mulnaes D, Stalke A, Dröge C, et al. Vasor: Accurate prediction of variant effects for amino acid substitutions in multidrug resistance protein 3. Hepatol Commun. 2022;6(11):3098-111.

5. Gonzales E, Gardin A, Almes M, Darmellah-Remil A, Seguin H, Mussini C, et al. Outcomes of 38 patients with PFIC3: Impact of genotype and of response to ursodeoxycholic acid therapy. JHEP Rep. 2023;5(10):100844.

6. Sharma A, Poddar U, Agnihotry S, Phadke SR, Yachha SK, Aggarwal R. Spectrum of genomic variations in Indian patients with progressive familial intrahepatic cholestasis. BMC Gastroenterol. 2018;18(1):107.

7. Jaganathan K, Kyriazopoulou Panagiotopoulou S, McRae JF, Darbandi SF, Knowles D, Li YI, et al. Predicting Splicing from Primary Sequence with Deep Learning. Cell. 2019;176(3):535-48.e24.

8. Yeo G, Burge CB. Maximum entropy modeling of short sequence motifs with applications to RNA splicing signals. J Comput Biol. 2004;11(2-3):377-94.

9. Richards S, Aziz N, Bale S, Bick D, Das S, Gastier-Foster J, et al. Standards and guidelines for the interpretation of sequence variants: a joint consensus recommendation of the American College of Medical Genetics and Genomics and the Association for Molecular Pathology. Genet Med. 2015;17(5):405-24.

10. Tavtigian SV, Harrison SM, Boucher KM, Biesecker LG. Fitting a naturally scaled point system to the ACMG/AMP variant classification guidelines. Hum Mutat. 2020;41(10):1734-7.

11. ClinGen. ClinGen Variant Classification Guidance [Available from: <https://clinicalgenome.org/tools/clingen-variant-classification-guidance/>.
